# A New Triterpene from the Plant of *Uncaria Macrophylla*

**DOI:** 10.3390/molecules17010504

**Published:** 2012-01-05

**Authors:** Guangli Sun, Xiaopo Zhang, Xudong Xu, Junshan Yang, Mingliang Zhong, Jingquan Yuan

**Affiliations:** 1 Institute of Medicinal Plant Development, Chinese Academy of Medical Sciences & Peking Union Medical College, Beijing 100193, China; Email: guanglisun@126.com (G.S.); xiaopozhang2011@126.com (X.Z.); junshanyang@sina.com (J.Y.); mingliangzhongsky@163.com (M.Z.); yjqgx@163.com (J.Y.); 2 Department of Pharmacy, School of Pharmacy, Hebei United University, Tangshan, Hebei 063000, China; 3 Guangxi Institute of Medicinal Plant Development, Nanning, Guangxi 530023, China

**Keywords:** triterpene, 3β,6β,19α-trihydroxy-urs-12-en-28-oic acid-24-carboxylic acid methyl ester, *Uncaria macrophylla*, Rubiaceae, antitumor activity

## Abstract

Our ongoing investigations on the stem bark of *Uncaria macrophylla* afforded a new ursolic triterpene, 3β,6β,19α-trihydroxy-urs-12-en-28-oic acid-24-carboxylic acid methyl ester (**1**), named uncariursanic acid, and three known ursolic triterpenes including 3β,6β,19α-trihydroxy-23-oxo-urs-12-en-28-oic acid (**2**), 3β,6β,19α-trihydroxy-urs-12-en-28-oic acid (**3**) and ursolic acid (**4**). Their structures were elucidated by extensive spectral methods, including 1D and 2D NMR and HR-ESI-MS. The cytotoxicities of the four compounds were evaluated against two cancer cell lines (MCF-7 and HepG2) by the MTT method, and only compound **4** exhibited potent activity.

## 1. Introduction

Uncaria, an important source of medicinal natural products from the Rubiaceae family, is widely distributed in tropical regions, including Southern Asia, Africa and South America [1]. Many species of the *Uncaria* genus have been broadly used as drugs for curing wounds, ulcers, fevers, headaches, hypertension, and gastrointestinal illness [2,3]. Many studies concerning the chemical constituents of Uncaria species have been carried out, which have led to the isolation of a series of secondary metabolites including alkaloids, triterpenes and flavones [4,5,6,7]. In our ongoing research on the systematic isolation of phytochemical constituents, four triterpenes were isolated. Their structures have been identified by extensive spectral methods including 1D, 2D NMR and MS. This paper mainly deals with the isolation, structural elucidation of the new compound, as well as the cytotoxicities of the four triterpenes.

## 2. Results and Discussion

**Figure 1:**
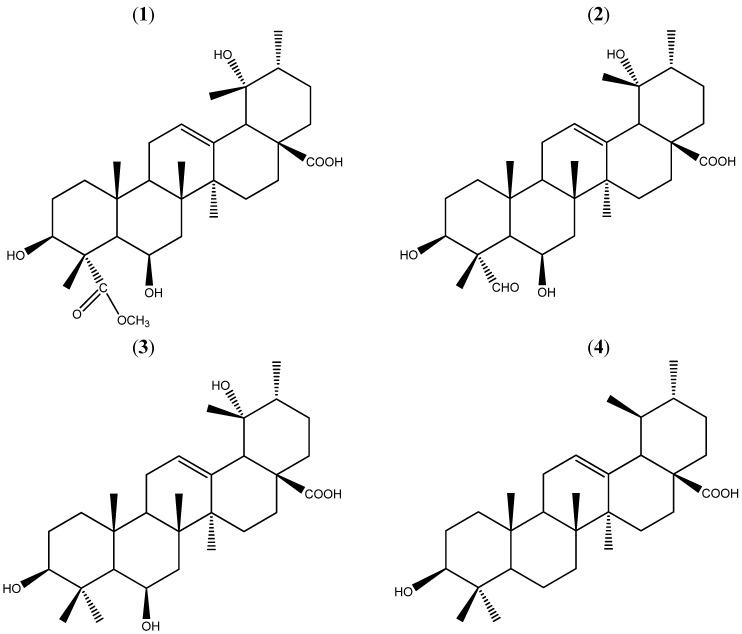
Structures of **1**–**4**. Compounds **1**–**4** were isolated from the chloroform extracts.

Compound 1 (Figure 1) was isolated as a white solid from the chloroform extracts of *Uncaria macrophylla*. The HR-ESI-MS spectrum revealed a quasi-molecular ion peak at 555.3281 [M+Na]^+^ (calculated 555.3298) corresponding to the molecular formula of C_31_H_48_O_7_Na. [α]^25^_D_ +4.86°.

The ^1^H-NMR spectral data showed peaks at δ 1.07 (3H, s), 1.20 (3H, s), 1.32 (3H, s), 1.33 (3H, s), 1.48 (3H, s) and δ 0.93 (3H, d, *J* = 7.2 Hz) due to five methyl groups on quaternary carbons and one methyl group on a tertiary carbon. In the ^13^C APT spectrum, signals of the methyl groups at δ 18.65, 27.27, 25.03, 17.63, 12.88 and 16.77 were clearly observed. An signal oleﬁnic proton at δ 5.32 in the ^1^H-NMR spectrum along with signals at δ 129.72 and 139.51 in the ^13^C-NMR spectrum suggested **1** was a characteristic ursolic pentacyclic triterpene posessing a Δ^12,l3^ moiety [8]. Further, signals at δ 180.49 and 182.52 in the ^13^C-NMR spectrum together with the IR absorption at 1711 cm^−1^ suggested the existence of two carboxyl groups in **1**. The ^1^H-NMR signals at δ 3.90 and δ 3.84 and ^13^C-NMR peaks at δ 77.40, 72.06 and 73.82 suggested **1** to be a trihydroxy-substituted pentacyclic triterpene.

**Figure 2:**
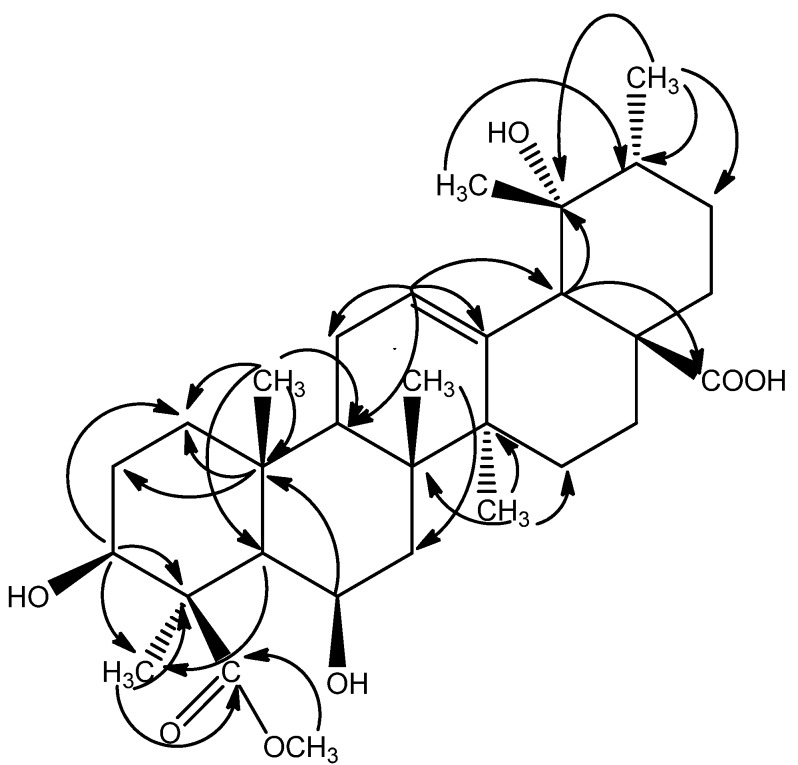
Selected HMBC (^2^J and ^3^J) correlations of compound **1**.

The signal at δ 3.69 (3H, s) in the ^1^H-NMR spectrum and the signal at δ 52.59 in the ^13^C-NMR spectra were due to a methoxyl group attached to a carbonyl group, which was confirmed by the correlation between H-31 (δ 3.69) and C-24 (δ 180.49) in the HMBC experiment. The Δ^12, l3^ structure was also confirmed by the HMBC correlations between H-12 (δ 5.32) and C-9 (δ 49.40), C-11 (δ 24.80), C-13 (δ 139.51), C-18 (δ 55.36). The HMBC cross-peaks between H-3 (δ 3.90) and C-1 (δ 42.00), between H_3_-25 and C-1 (δ 42.00), C-5 (δ 54.51), C-9 (δ 49.40), C-10 (δ 37.42), and the evidence from the chemical shift and the *J* value of the axial proton at C-3 (δ 3.90, dd, *J* = 12.0, 3.6 Hz, H-3) suggested the hydroxy at C-3 was in a β-configuration. HMBC correlations between H-6 (δ 3.84) and C-7 (δ 42.03), C-10 (δ 37.42) indicated a hydroxyl group was attached to the C-6 (Figure 2).

**Figure 3:**
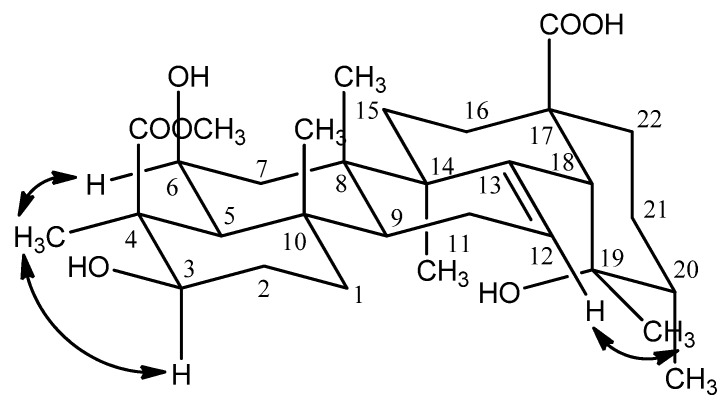
Selected NOESY correlations of compound **1**.

The relative configuration of the hydroxyl group at C-6 was deduced to be β by the cross-peaks of H-3 (δ 3.90) and H_3_-23 (δ1.48), H-6 (δ 3.84) and H_3_-23 (δ 1.48) in the NOESY experiment (Figure 3). The hydroxyl group at the 19 position induced a downfield shift of the resonance of the axial proton at C-16. This signal resonated at δ 2.55 and was observed as a ddd with *J* = 13.2, 13.2, 4.8 Hz, thus supporting the 19α-OH stereochemistry and being compatible only with a *cis* stereochemistry of the ring D/E junction; the proton at δ 2.55 was conﬁrmed to be H-16 [5]. The appearance of characteristic signals at δ 2.52 (1H, s, H-18) in the ^1^H-NMR spectrum and at δ 73.82 ppm (C-19, quaternary carbon) in the ^13^C-NMR spectrum suggested that **1** was an ursolic acid derivative with a hydroxyl group attached to C-19, which was confirmed by the observed correlation between H-18 and C-14 (δ 43.28), C-16 (δ 26.82), C-19 (δ 73.82), C-28 (δ 182.52), between H_3_-29 (δ 1.20) and C-18 (δ 55.36), C-20 (δ 43.30), between H_3_-30 (δ 0.93) and C-19 (δ 73.82) in the HMBC experiment (Figure 2). Furthermore, the NOESY correlations between H-12 (δ 5.32) and H-29 (δ 1.20) confirmed that the 19-OH accepted α configuration (Figure 3).

Consequently, the structure **1** was established and determined to be 3β,6β,19α-trihydroxy-urs-12-en-28-oic acid-24-carboxylic acid methyl ester (Figure 1), and compound **1** was named uncariursanic acid. The structures of the three known compounds **2**–**4** were identified by the ^1^H-NMR and ^13^C-NMR spectral data, which were consistent with the published data [9,10].

The four triterpenes were evaluated for their *in vitro* inhibitory abilities against two cancer cell lines (HepG2 and MCF-7). The results showed that only ursolic acid and *cis*-platin were effective in inhibiting the growth of HepG2 and MCF-7. The IC_50_ values of ursolic acid against the two cell lines were 12.1 µg/mL and 15.1 µg/mL and *cis*-platin were 7.5 µg/mL, 8.2 µg/mL, respectively. However, compounds **1**–**3** showed no inhibitive activities against the two cancer cell lines in the experiment (IC_50_ > 100 µg/mL).

## 3. Experimental

### 3.1. General

Infrared spectra were measured using a FTIR-8400S spectrometer. The HR-ESI-MS data were obtained on a Micross Mass Autospec-Ultima ETOF mass spectrophotometer (Waters Ltd., England). NMR spectra were measured on a Bruker AV-600 (600 MHz for ^1^H and 150 MHz for ^13^C) spectrometer (Bruker Biospin Inc., Germany) using CD_3_OD as solvent and tetramethylsilane (TMS) as internal standard. Ultraviolet spectra were recorded in MeOH on a Shimadzu UV-160A, UV-Visible Recording Spectrophotometer (Shimadzu Corporation, Japan). ODS gel (40–60 μm, Daiso Co., Ltd., Japan). Chromatography was performed on silica gel (200–300 mesh, Qingdao Haiyang Chemical Factory, Qingdao, China).

### 3.2. Plant Material

The medicinal material was collected from Guangxi Province in 2010, and identified by Dr. Jing Quan Yuan at the Institute of Medicinal Plant Development, Chinese Academy of Medical Sciences & Peking Union Medical College, where a voucher specimen with No.20101132 has been deposited.

### 3.3. Extraction and Isolation

The air-dried and powdered sample (5 kg) was extracted successively with 90% EtOH-H_2_O (50 kg, 70 °C, 2 h). The extract was filtered and concentrated under reduced pressure until only H_2_O remained. The remaining solution was sequentially partitioned with petroleum ether (boiling point 60–90 °C), CHCl_3_ and EtOAc, to obtain petroleum ether (32.3 g), chloroform (31.8 g), ethyl acetate (15.6 g) and water (8.5 g) extracts after concentration. The chloroform extract was chromatographed over a silica gel column using a stepwise gradient system of petroleum ether/acetone. Eluted fraction A (petroleum ether/acetone 70:30, 3.2 g) was then subjected to Sephadex LH 20 (Pharmacia) column chromatography using chloroform /methanol (40:60), the obtained fraction C (200 mg) was subjected to reverse phase chromatography (ODS) with MeOH-H_2_O (40:60) to give compound **1** (5 mg), compound **2** (16 mg), compound **3** (12 mg) and compound **4** (10 mg).

### 3.4. Spectral Data

*3β,6β,19α-Trihydroxy-urs-12-en-28-oic acid-24-carboxylic acid methyl ester* (**1**). White solid. IR ν_max_ (cm^−1^): 3461, 2931, 1711. ESI-MS *m/z*: [M+Na]^ +^ 555. For ^1^H-NMR and ^13^C-NMR (CD_3_OD) spectra, see Table 1.

**Table 1:** ^1^H-NMR and ^13^C-NMR data for **1**.

Position	^1^H(δ)	^13^C(δ)	HMBC
1	1.63 (m, 2H)	42.00	37.42 (C-10)
2	1.60 (m, 2H)	27.74	37.42 (C-10)
3	3.90 (dd, 1H, 3.6, 12 Hz)	77.40	42.00 (C-1), 56.42 (C-4), 12.88 (C-23), 180.49 (C-24)
4	-	56.42	
5	1.50 (m, 1H)	54.15	12.88 (C-23)
6	3.84 (m, 1H)	72.06	
7	1.46 (m, 1H),1.69 (m, 1H)	42.03	
8	-	40.93	
9	1.78 (m, 1H)	49.40	
10	-	37.42	
11	2.05 (m, 2H)	24.80	
12	5.32 (t, 1H, 3.6 Hz)	129.72	49.40 (C-9), 24.80 (C-11), 139.51 (C-13), 55.36 (C-18)
13	-	139.51	
14	-	43.28	
15	0.98 (m, 1H), 1.86 (ddd,1H, 13.2, 13.2, 4.8 Hz)	29.78	
16	1.51 (m, 1H), 2.55 (ddd,1H, 13.2, 13.2, 4.8 Hz)	26.82	29.78 (C-15), 49.26 (C-17)
17	-	49.26	
18	2.52 (s, 1H)	55.36	43.28 (C-14), 26.82 (C-16), 73.82 (C-19), 182.52 (C-28)
19	-	73.82	
20	0.94 (1H)	43.30	
21	1.73 (m, 2H)	27.49	
22	1.73 (m, 2H)	39.18	
23	1.48 (s, 3H)	12.88	56.42 (C-4), 180.49 (C-24)
24	-	180.49	
25	1.33 (s, 3H)	17.63	42.00 (C-1), 54.15 (C-5), 49.40 (C-9), 37.42 (C-10)
26	1.07 (s, 3H)	18.65	42.03 (C-7), 40.93 (C-8), 49.40 (C-9), 43.28 (C-14)
27	1.32 (s, 3H)	25.03	40.93 (C-8), 43.28 (C-14), 29.78 (C-15)
28	-	182.52	
29	1.20 (s, 3H)	27.27	55.36 (C-18), 43.30 (C-20)
30	0.93 (s, 3H)	16.77	73.82 (C-19), 43.30 (C-20), 27.49 (C-21)
31	3.69 (s, 3H)	52.59	180.49 (C-24)

*3β,6β,19α-Trihydroxy-23-oxo-urs-12-en-28-oic acid* (**2**). White solid. IR ν_max_ (cm^−1^): 3419, 2930, 1709, 1689. ESI-MS *m/z*: [M+Na]^+^ 525. The ^1^H-NMR and ^13^C-NMR (CD_3_OD) spectral data were consistent with the published data [9].

*3β,6β,19α-Trihydroxy-urs-12-en-28-oic acid* (**3**). White solid. IR ν_max_ (cm^−1^): 3437, 2931, 1688. ESI-MS *m/z*: [M+Na]^ +^511. The ^1^H-NMR and ^13^C-NMR (CD_3_OD) spectral data were consistent with the published data [9].

*Ursolic acid* (**4**). White solid. IR ν_max_ (cm^−1^): 3422, 2927, 1693. ESI-MS m/z: [M+Na]^+^ 479. The ^1^H-NMR and ^13^C-NMR (pyridine-d_5_) spectral data were consistent with the published data [10].

### 3.5. Bioassays

Antitumor activity was assayed on HepG2 liver tumor cells and MCF-7 breast tumor cells using *cis*-platin as positive control. Cells were plated in the appropriate media on 96-well plates in a 100 μL total volume at a density of 6 × 104 cells/mL. The final concentrations of each compound were 0.625, 1.25, 2.5, 5.0, 10 μg/mL. The plates were incubated at 37 °C in 5% CO_2_ for 72 h. Cell viability was determined based on the mitochondrial conversion of MTT to formazan.

## 4. Conclusions

A new triterpene, 3β,6β,19α-trihydroxy-urs-12-en-28-oic acid-24-carboxylic acid methyl ester (**1**), along with three known triterpenes were isolated from the stem bark of *U. macrophylla.* The isolation of the new compound was a new addition to the molecular diversity of *U. macrophylla.* Ursolic acid (**4**) exhibited antitumor activity.
